# A study on Shine-Muscat grape detection at maturity based on deep learning

**DOI:** 10.1038/s41598-023-31608-6

**Published:** 2023-03-20

**Authors:** Xinjie Wei, Fuxiang Xie, Kai Wang, Jian Song, Yang Bai

**Affiliations:** 1 College of Mechanical and Electronic Engineering, Shandong University of Science and Technology, Qingdao, 266590 Shandong China; 2 School of Mechanics and Automation, Weifang University, Weifang, 261061 Shandong China

**Keywords:** Mechanical engineering, Engineering, Physics

## Abstract

The efficient detection of grapes is a crucial technology for fruit-picking robots. To better identify grapes from branch shading that is similar to the fruit color and improve the detection accuracy of green grapes due to cluster adhesion, this study proposes a Shine-Muscat Grape Detection Model (S-MGDM) based on improved YOLOv3 for the ripening stage. DenseNet is fused in the backbone feature extraction network to extract richer underlying grape information; depth-separable convolution, CBAM, and SPPNet are added in the multi-scale detection module to increase the perceptual field of grape targets and reduce the model computation; meanwhile, PANet is combined with FPN to promote inter-network information flow and iteratively extract grape features. In addition, the CIOU regression loss function is used and the prior frame size is modified by the k-means algorithm to improve the accuracy of detection. The improved detection model achieves an AP value of 96.73% and an F1 value of 91% on the test set, which are 3.87% and 3% higher than the original network model, respectively; the average detection speed under GPU reaches 26.95 frames/s, which is 6.49 frames/s higher than the original model. The comparison results with several mainstream detection algorithms such as SSD and YOLO series show that the method has excellent detection accuracy and good real-time performance, which is an important reference value for the problem of accurate identification of Shine-Muscat grapes at maturity.

## Introduction

Grapes are one of the major fruits in the world, while China is also a major country for growing grapefruit trees. Shine-Muscat grapes (Vitis labruscana Bailey × V. vinifera L.), also known as Xayin Muscat, Jinhua Rose, and Bright Rose, are European and American hybrids that ripen similarly to “Giant Peak”, with yellow-green skin, thin skin, and hard, crisp flesh, and are especially known for their rich rose It is known for its strong rose flavor^1^. Currently, most of the Shine-Muscat grapes in China still need to be picked manually, which requires a lot of labor and leads to high production costs in orchards. To achieve automated harvesting of sun rose grapes, the primary problem to be solved is to identify and locate fruit targets quickly and accurately. Efficient detection of target fruits in natural environment is one of the key technologies for fruit estimation, precision agriculture and mechanized automatic picking. Grape detection models based on traditional algorithms do not balance detection accuracy and real-time well, and cannot meet the needs of picking robots for rapid identification and localization of grape fruits in orchards. Therefore, using deep learning to quickly and accurately detect Shine-Muscat grapes in orchards has significant application value and important practical significance for the development of grape picking robots^2^.

In recent years, many domestic and foreign researchers have proposed different target detection and identification algorithms for detecting orchard grapes^2–8^. Ping Liu et al.^9^ used the H component in HSV color space to obtain the overlapping grape bunch region, used the slope between the inflection point and the center point to determine the exact location of the grape bunches, and then used the Chan_Vese model to identify the grape bunches, and finally fused the overlapping boundary contours and image contours to identify the grape bunches, and the average accuracy of this method was 89.71%, and the recognition success rate reached 90.91%. Cha et al.^10^ compared five transfer learning models to recognize grape clusters with different light intensities and different maturity levels, and found by comparison that the VGG16 transfer learning network model had the best recognition results compared to the network structures of Resnet50, GoogleNet, VGG19 and AlexNet, and the average accuracy of model recognition The average accuracy of the model recognition reached 99.07% and the average detection time was 26 ms, which achieved better recognition of grape clusters in the orchard environment, but failed to meet the requirement of real-time detection. Thiago et al.^11^ detected grape clusters in the publicly available dataset WGISD by using the Mask R-CNN algorithm and achieved an F1 score of 0.840 with IOU equal to 0.5, but the algorithm was tedious and time-consuming to label the dataset and had an average real-time detection performance. Xiang-Yu Cui et al.^12^ used trainable transformers and multiscale feature map fusion for YOLO algorithm improvement to achieve the task of detecting grapes, and by self-designed feature extraction network and loss function made the F1 score of this network model reach 92.58% on the test set, which achieved a better detection effect, but training this network cannot use migration learning and the training consumes a long time. Reis et al.^13^ developed a visual detection recognition grape system that was able to automatically distinguish between white and red grapes and achieved a better classification task for both grapes, achieving 97% and 91% correct classification results, respectively. Liu et al.^6^ used support vector machines to accelerate grape bunch detection by combining color and texture information to process images, with detection accuracy and recall of 88% and 91.6% for two red grape varieties (Shiraz and Cabernet Sauvignon), but red grape varieties interfered less with detection and were not tested for grapes with similar background color varieties were tested. Lu et al.^14^ proposed a wine grape bunch detection model by combining YOLOv5 with Swin-transformer to detect both Chardonnay and Merlot grapes, and the experimental results were able to achieve good detection results for Chardonnay grapes, but poor performance for Merlot grapes. In addition, with the continuous development of deep learning, deep learning-based target detection algorithms have been applied to other fruit detection^15–17^. Long sheng Fu et al.^18^ used depth features to filter background objects to improve the accuracy of apple detection by first using a depth filter to remove background trees and then using the Faster R-CNN algorithm to detect apples, and the method improved the recognition accuracy by 2.5%.Dandan Wang et al.^19^ used the channel pruning algorithm to modify the YOLOv5 model, and the accuracy, recall, F1 score, and false detection rate of the modified model were 95.8%, 87.6%, 91.5%, and 4.2%, respectively, to achieve fast detection of target apples. Gai et al.^20^ added DenseNet to the YOLOv4 backbone feature extraction network to detect cherry fruit, increasing the feature extraction capability, and the F1 score of this model reached 0.856 on the test set, but the long-range detection was poor. Chen et al.^21^ improved the YOLOv3 model for detecting cherry tomatoes using a dual-path network^22^ for feature extraction and achieved multi-scale detection by creating four feature layers at different scales, with a model detection accuracy of 94.29%.

To balance the high accuracy and detection speed of the grape detection algorithm, as well as to achieve efficient automated machine picking of the Shine-Muscat grapes, the use of YOLO family models in orchards has also become a current research hotspot, so this study proposes a grape detection model based on improved YOLOv3^23^, which improves the backbone feature extraction network (Backbone), a multiscale detection module, and loss function of YOLOv3 algorithm, making this target detection algorithm with high detection accuracy and detection speed, which can be applied to fast visual detection of machine picking of the Shine-Muscat grapes in orchards.

## Related works

### Shine-Muscat grape dataset

**Figure 1:**
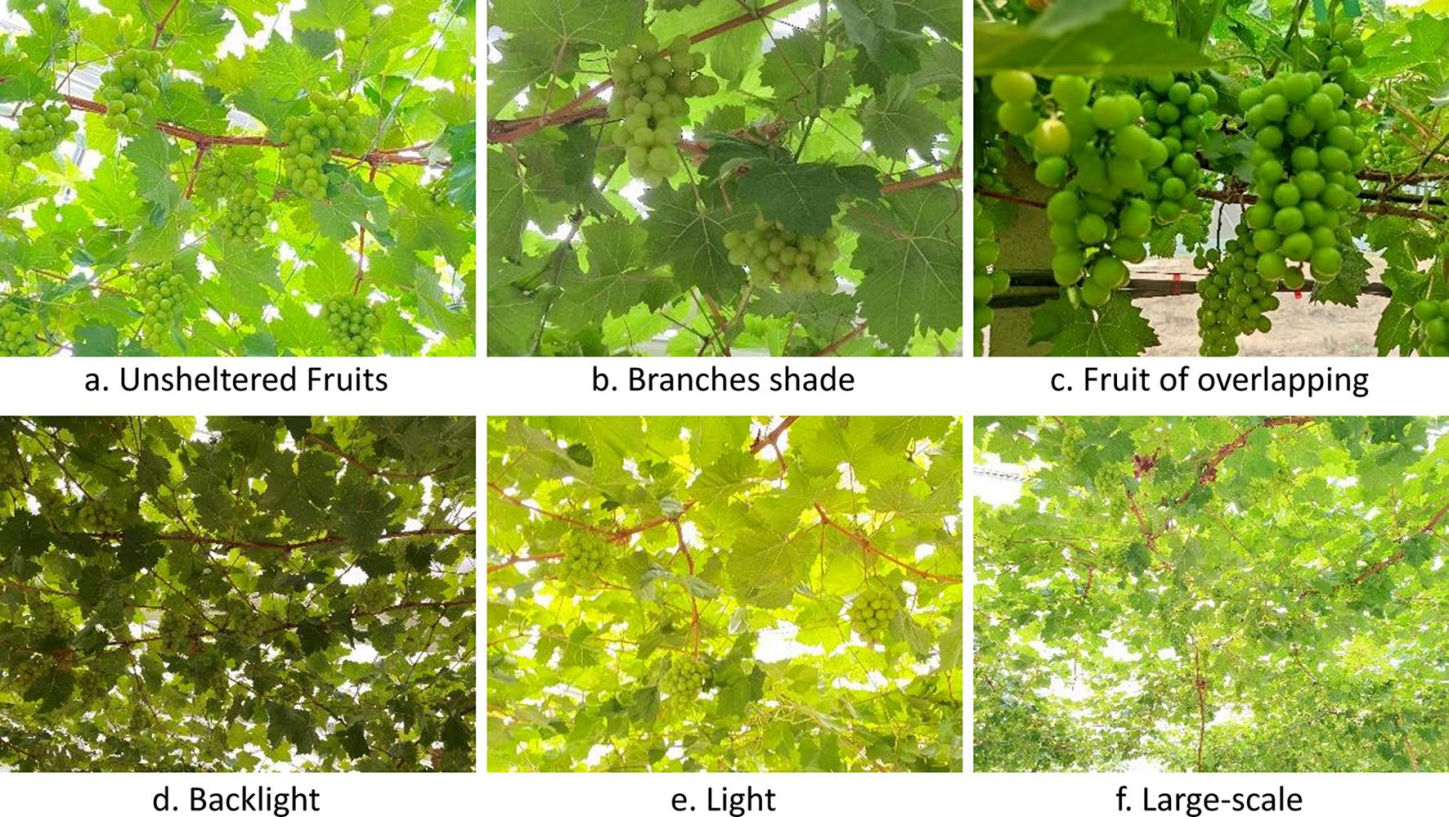
Images of shine-muscat grape under different environmental conditions.

This subsection describes the grape dataset used for the study and the data enhancement of the images. The address of the image dataset collected for this study was located at Xiang Yun Grape Horticulture Farm, Shou guang City, Shandong Province (Cooperative of Institute of Botany, Chinese Academy of Agricultural Sciences), and the collection time was from 10:00 a.m. to 12:00 p.m. on January 7, 2022 (sunny day), and the grape variety was Shine-Muscat, and the Shine-Muscat grape berries were collected at maturity. Photographs were taken using a Redmi k30 Ultra type smartphone at a distance of 0.5–1.5 m from the grapes to collect fruit under a branch and leaf shading, overlapping fruit, down light and backlight to ensure sample diversity. After flipping, scaling and other data enhancement means to obtain a total of 1200 images, including 302 unobscured fruit, 216 branches and leaves obscured, 245 fruit overlapping, 123 large scene images, 148 smooth light, and 166 backlight, divided into a training set and test set according to the ratio of 8:2, including 960 training set and 240 validation set. The images were labeled using LabelImg software and the image information was saved in PASCAL VOC dataset format with original image resolution sizes of 3472 × 4624 pixels and 4624 × 3472 pixels. The information of the training dataset is shown in Table 1. Some of the grape dataset images are shown in Fig. 1, which contains grape images under different environmental conditions.

**Table 1:** Information on the shine-muscat grape dataset.

Different environment	Unsheltered fruits	Branches shade	Fruit of overlapping	Light	Backlight	Large-scale	Total number	Number of training set	Number of test set
Number of images	302	216	245	148	166	123	1200	960	240
Number of labeled grapes	1426	869	578	543	601	594	4611	3689	922

### Data enhancement

**Figure 2:**
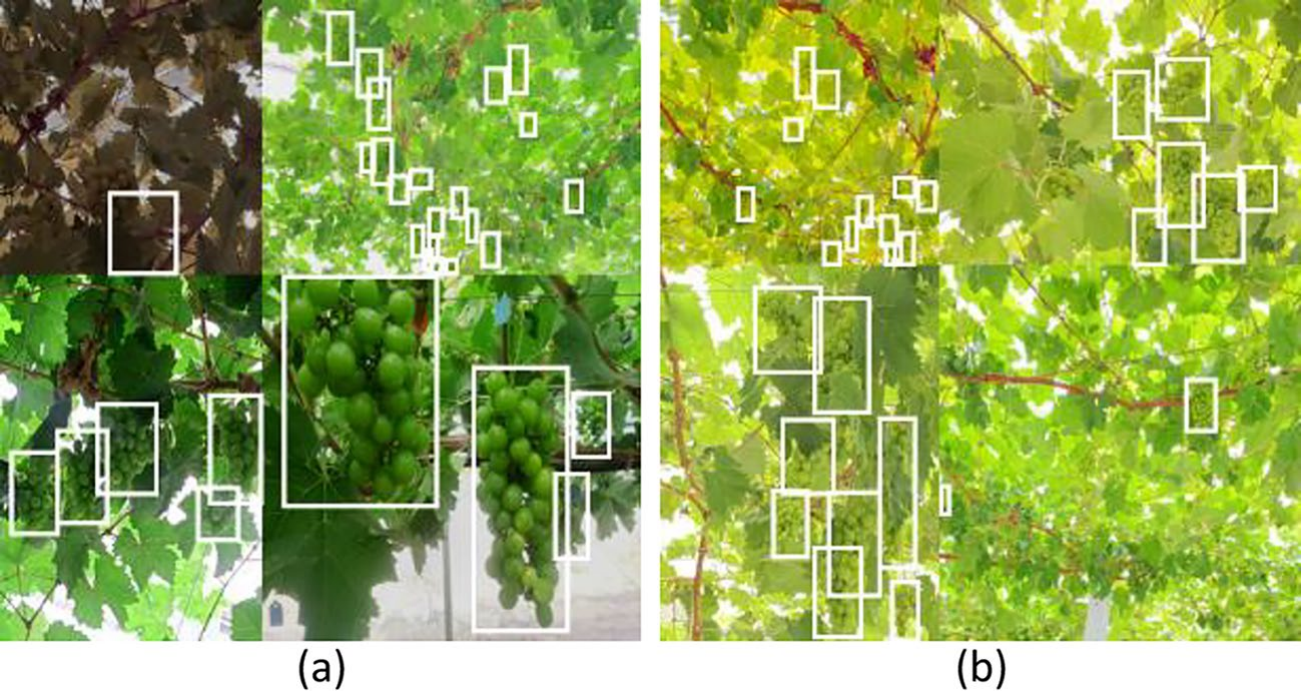
Mosaic data enhancement image.

The Mosaic^24^ data enhancement method is used in the image input network model for training, i.e., each training reads 4 images for a series of operations such as random scaling, flipping, cropping, and optical transformation, then, these 4 images are stitched together and the adjusted labels are passed into the network, which is equivalent to passing 4 images into the network for learning at the same time, largely enriching the detection object's The background information of the detected objects is largely enriched and the number of targets is increased, and the data of the four images will be calculated simultaneously in the normalized BN^25^ (Batch Normalization) calculation, which is equivalent to increasing the Batch size, making the mean and variance calculated in the BN layer more consistent with the distribution of the overall dataset and making the robustness of the model enhanced. Some of the images after Mosaic enhancement are shown in Fig. 2.

### Improvement based on yolov3 shine-muscat grape detection network

This subsection details the optimization and improvement of the YOLOv3 model, including three parts: the backbone feature extraction network, the multiscale detection module, and the localization loss function, and introduces the testbed and model hyperparameter settings. For the algorithm of fruit detection, the accuracy and real-time of the target detection algorithm should be considered to meet the needs of picking robots for efficient picking operations. To efficiently and accurately detect Shine-Muscat grapes in orchards, achieve efficient machine-picked grapes, and reduce the high cost caused by manual picking, this study proposes the S-MGDM (Shine-Muscat Grape Detection Model) grape detection model. The model first uses CSDenseNet, a network structure combining CSPNet^26^ and DenseNet^27^, as the backbone network for feature extraction, which makes the network detection speed increase significantly without basically affecting the detection accuracy, while reducing the network model and floating point operations, and combines the depth separable convolution^28^, SPPNet^29^, attention mechanism, and CIOU^30^ loss function to improve the multiscale detection module and loss function, respectively, to further improve the accuracy of grape fruit detection.

### Improvement of backbone feature extraction network

In this study, the improved YOLOv3 backbone feature extraction network CSDenseNet is used as the backbone network to accomplish the task of fast extraction of target features from grape images. The CSDenseNet network is based on the improvement of the DarkNet53^23^ network, and the CSPNet module and DenseNet module are introduced for the problem of cluster adhesion and green branch occlusion with a similar color to the fruit in Shine-Muscat grapes so that more useful underlying grape information can be extracted to improve the accuracy of identifying grape fruit, and the number of floating point operations (FLOPs) of the model is effectively reduced to ensure both the speed and accuracy of inference and to reduce the computational effort of the model.

**Figure 3:**
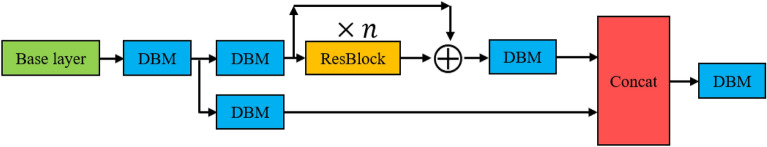
Structure of Resblock.

**Figure 4:**
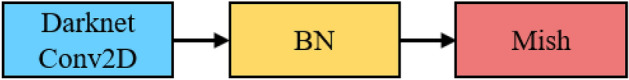
DBM module.

CSPNet (Cross Stage Partial Network) is a method to solve the problem of duplication of gradient information in network optimization by separating the gradient streams and making them propagate on different network paths, avoiding the reuse of gradient information and drastically reducing the number of floating-point operations (FLOPs) of the network model. (FLOPs) of the network model, which ensures the speed and accuracy of inference while also reducing the computational effort of the model. Therefore, in this paper, the residual blocks in DarkNet53 are combined with CSPNet as the Resblock structure, as shown in Fig. 3. The module first compresses the input feature map in height and width through the first DBM module, which is shown in Fig. 4 and consists of Conv2D, BN and Mish activation functions. The main part continues the stacking of the original residual blocks, and the other part builds a large residual edge, which bypasses multiple residual structures, and then Concatenate splices these two parts, and finally integrates the number of channels through a DBM module.

**Figure 5:**
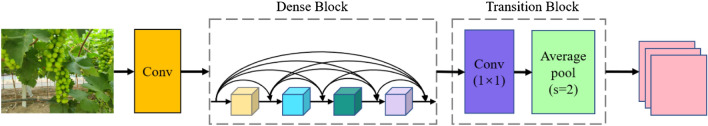
DenseNet network structure.

The module first compresses the input feature map in height and width through the first DBM module, which is shown in Fig. 5 and consists of Conv2D, BN, and Mish activation functions. The main part continues the stacking of the original residual blocks, and the other part builds a large residual edge, which bypasses multiple residual structures, and then Concatenates splices these two parts, and finally integrates the number of channels through a DBM module. The DenseNet backbone network establishes connections between the layers, making full use of the image feature information and ensuring efficient use of the feature maps used by the target detection branch network.

**Figure 6:**
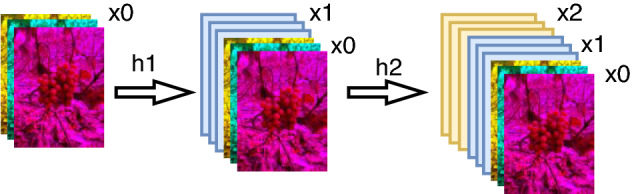
Diagram of DenseBlock.

As shown in Fig. 6, in DenseBlock, the feature maps of each layer are of the same size and can be stitched together in the channel dimension. The non-linear combinatorial function in the DenseBlock module adopts the BN + ReLU + Conv structure. First, a 1 × 1 convolution is performed on the input feature layer to adjust the number of channels to obtain c feature maps in order to reduce the number of features and thus improve computational efficiency; The feature map of $k$ channels is then obtained using 3 × 3 convolution, at which point, a feature layer with shape $\left( {h,w,k} \right)$ is obtained. Note that, unlike the ResNet^31^ structure, all layers in the DenseBlock output c feature maps after convolution, i.e., the number of channels of the resulting feature maps is c, and c is a hyperparameter. The c = 32 used in this study gave better performance in the experiment. Assuming that the number of channels in the input feature map is $k_{0}$, then the number of channels in the c-layer input is $k_{0} + k(l - 1)$. Thus, as the number of layers increases, although $k$ is set smaller, the DenseBlock will have more inputs as a result of feature reuse, and each layer has $k$ features unique to itself. After repeated splicing operations in the channel dimension, the original features are always preserved, as are the features, after convolutional processing. As the network gets deeper, a dense concatenation of all preceding and following layers can be achieved.

For the Transition Block module, its main function is to connect two adjacent Dense Block modules before and after each other, while reducing the size of the feature map. The Transition structure consists of a 1 × 1 convolution and a 2 × 2 AveragePooling2D^32^ structure to reduce the size of the feature map. If the DenseBlock connected in front of the Transition Block layer gets a feature map with channel number $m$, the Transition Block structure will have $\alpha m$ features, where c is the compression rate. When $\alpha$ = 1, the number of feature maps does not change through the Transaction Block layer, and $\alpha$ = 0.5 is used in this paper, i.e., the number of output channels is equal to half the number of feature map channels obtained from the previous DenseBlock.

The structure of the backbone feature extraction network used in this study is shown in Table 2. First, the input image goes through a DBM operation to obtain a 416 × 416 × 32 feature map, and then the obtained feature map goes through two Resblock modules to obtain a 104 × 104 × 128 feature map, and then three Denseblock modules and Transition Block The three Denseblock modules and the Transition Block module are used to obtain three feature maps of size 52 × 52 × 256, 26 × 26 × 512, and 13 × 13 × 1024, respectively, and the three feature maps are then passed into the enhanced feature extraction section for enhanced feature extraction.

**Table 2:** Backbone feature extraction network structure parameters.

Size of input	Operators	Size of output	Times
416 × 416 × 3	DBM	416 × 416 × 32	1
416 × 416 × 32	Resblock	208 × 208 × 64	1
208 × 208 × 64	Resblock	104 × 104 × 128	2
104 × 104 × 128	Denseblock	104 × 104 × 256	4
104 × 104 × 256	Transition Block	52 × 52 × 256	1
52 × 52 × 256	Denseblock	52 × 52 × 512	8
52 × 52 × 512	Transition Block	26 × 26 × 512	1
26 × 26 × 512	Denseblock	26 × 26 × 1024	16
26 × 26 × 1024	Transition Block	13 × 13 × 1024	1

### Multi-scale detection module improvements

**Figure 7:**
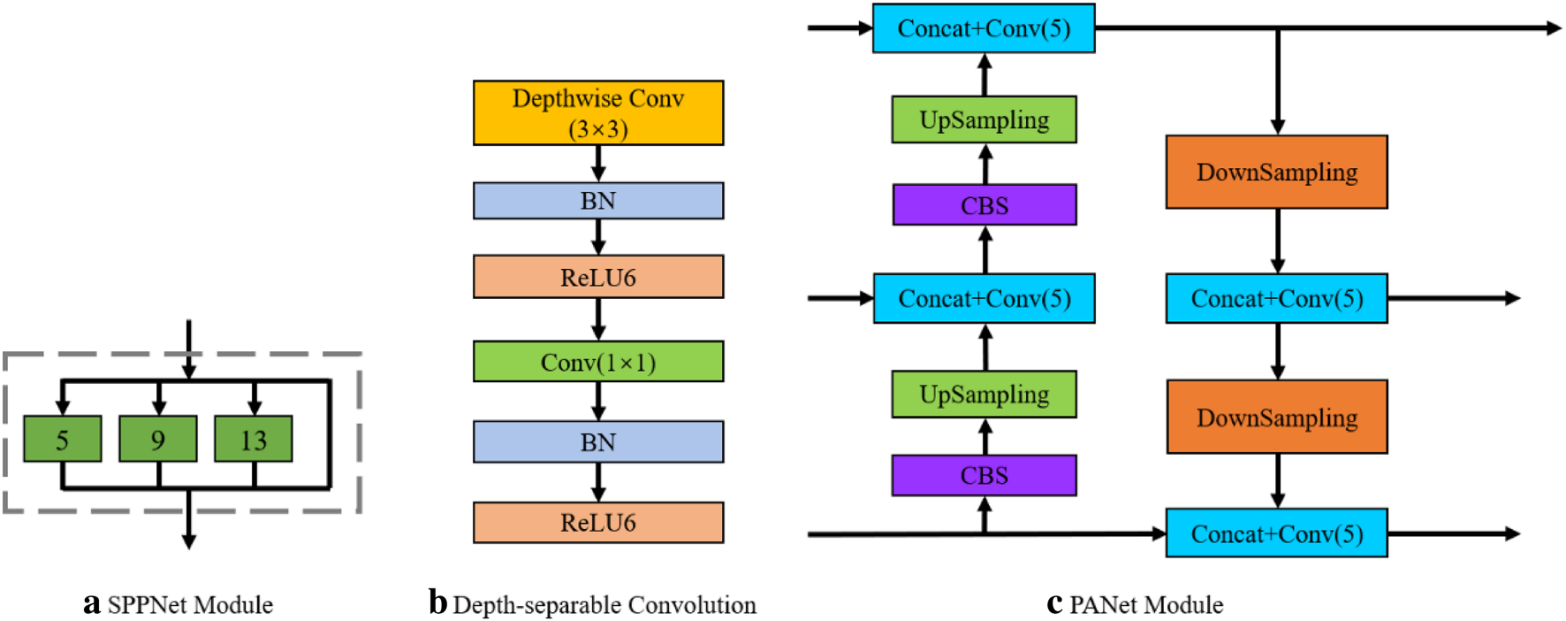
Structure of SPPNet, PANet and depth separable modules. *Note* CBS denotes the Conv + BN + SiLu structure, where SiLU is the combined Sigmoid and ReLU activation function; UpSampling means up-sampling operation, which increases the scale of the feature map by nearest interpolation in the width and height directions; DownSampling means down-sampling operation, which compresses the height and width of the feature map by depth-wise convolution with stride = 2; Depthwise Conv (3 × 3) means depth-wise convolution operation. Conv (1 × 1) indicates the number of channels adjusted by ordinary convolution, Same as below.

**Figure 8:**
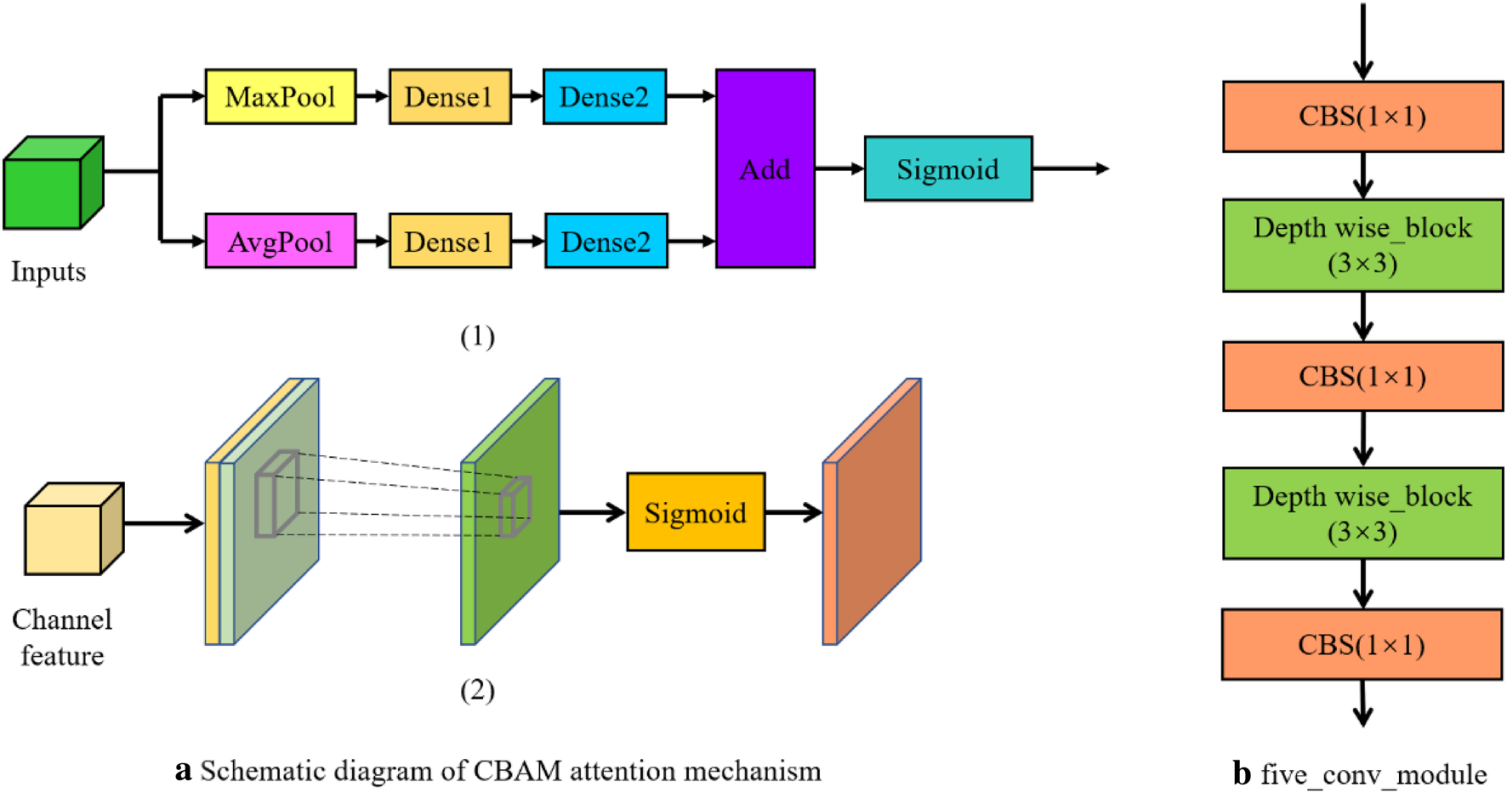
Attention mechanism and five_conv structure. *Note* A 3 × 3 depth separable convolution is used in the five_conv module to reduce the number of parameters.

To extract more high-level information of grapes, isolate significant target features as well as reduce the amount of parameter computation. This study draws on the ideas of SPPNet, PANet^33^, and depth-separable convolution, adding each of these modules to the part of enhanced feature extraction, and replacing the original structure with a modified multiscale detection module, making it possible to greatly reduce the number of parametric calculations based on accurate target detection. SPPNet, PANet, and depth-separable convolution modules are shown in Fig. 7a–c, respectively. In the improved multiscale detection module, the attention mechanism CBAM^34^ is added to the backbone feature extraction part after obtaining three feature maps of sizes 52 × 52 × 256, 26 × 26 × 512, and 13 × 13 × 1024, respectively, so that the network pays more attention to the channel and spatial information of grape The 13 × 13 × 1024 feature maps after adding the attention mechanism were first subjected to the five_conv operation for feature fusion, and then passed through the SPPNet structure to be processed using maximum pooling at four different scales, respectively, with maximum pooling kernel sizes of 13 × 13, 9 × 9, 5 × 5, and 1 × 1 (i.e., no operation), which can greatly increase the perceptual field and isolate the most significant contextual features of the grape target, and then pass the obtained output into the PANet module together with the two previous effective feature maps to achieve bottom-up and top-down operations as a way to iteratively extract grape features. The five_conv structure and the attention mechanism CBAM module are shown in Fig. 8a,b, where five_conv indicates that it consists of CBS(Conv + BN + SiLU) and Depthwise_block alternately repeated five times.

### Localization loss function improvement

**Figure 9:**
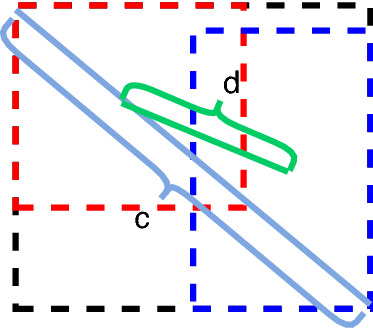
Diagram of CIOU.

The localization loss part of YOLOv3 still uses the sum-of-squares loss calculation method. Although this function takes into account the influence of different scales on the regression loss, using the sum-of-squares calculation method to calculate the x, y, w, and h directions separately will produce errors and does not take into account the correlation of location coordinates, and this calculation does not reflect well the degree of overlap between the network prediction frame and the actual real frame. Therefore, this study uses the CIOU loss function as an improved localization loss function. CIOU integrates the center distance between the target and anchor, overlap rate, scale, and penalty term, which makes the target frame regression become more stable, and the grape fruit target frame predicted by the network model is closer to the location and size of the real target frame, and does not, like IOU and GIOU^35^ problems such as divergence in the training process. And the penalty factor integrates the predicted frame aspect ratio to fit the target frame aspect ratio. The calculation formula is as follows.

1 $$ CIOU_{loss} = 1 - IOU + \frac{{\rho^{2} (b_{pred} ,b_{gt} )}}{{c^{2} }} + \alpha \nu $$

2 $$ \alpha = \frac{\nu }{(1 - IOU) + \nu } $$

3 $$ \nu = \frac{4}{{\pi^{2} }}\left( {\left( {\arctan \frac{{w^{gt} }}{{h^{gt} }}} \right) - \arctan \frac{w}{h}} \right)^{2} $$

where $\alpha$ is the weighting factor, and by definition the loss function is optimized in the direction of the larger overlap area, and $\nu$ measures the similarity of the network output prediction frame to the actual target frame aspect ratio. As shown in Fig. 9, $c$ is the length of the diagonal of the outer rectangle of the two boxes, and $d$ represents the Euclidean distance $\rho^{2} (b_{pred} ,b_{gt} )$ between the centers of the predicted and real boxes.

### Improved S-MGDM network architecture

**Figure 10:**
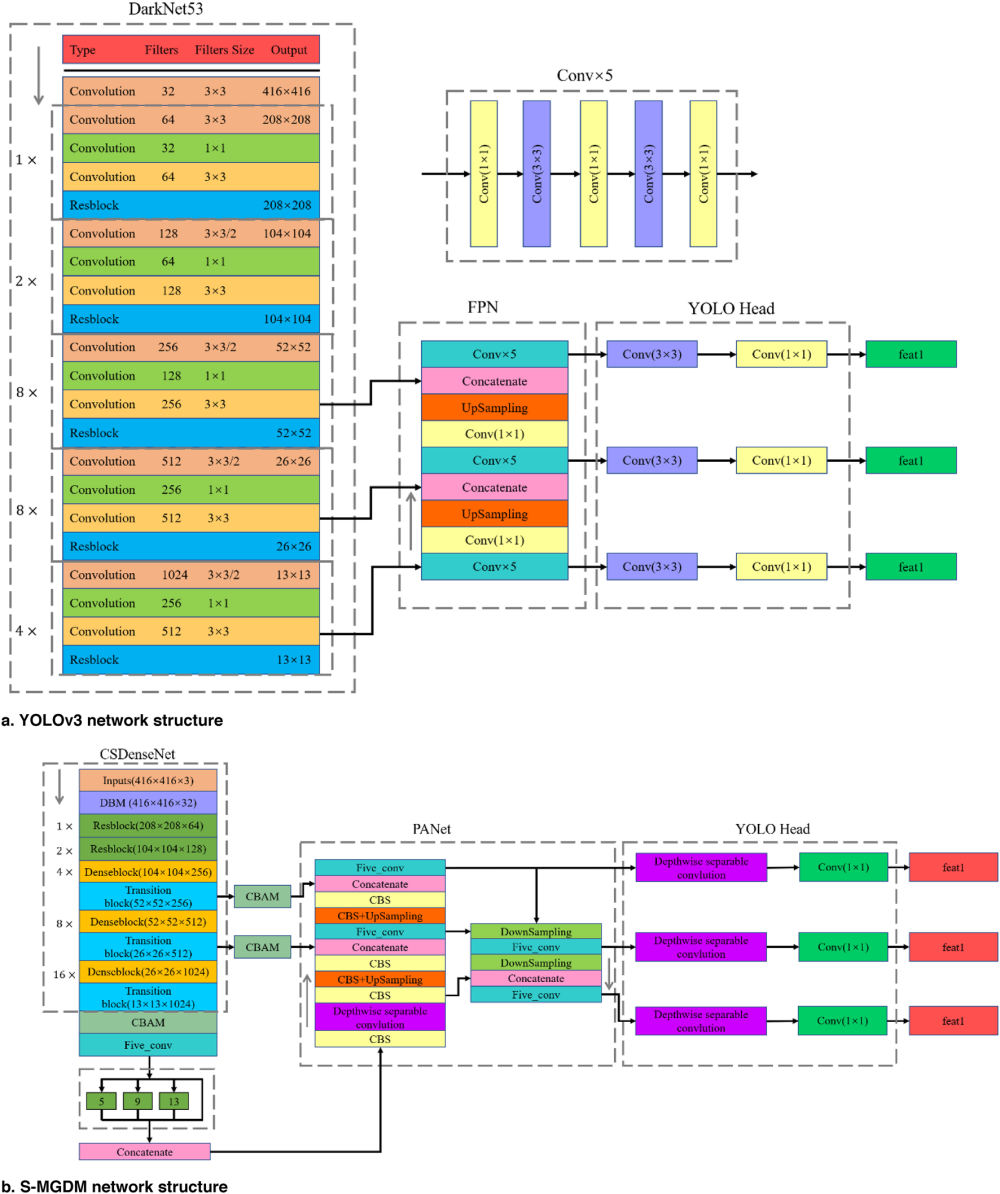
YOLOv3 and S-MGDM network architecture. *Note* Depthwise separable convolution means depthwise separable convolution operation, and fat1, fat2 and fat3 indicate the final output of the network respectively.

The S-MGDM network replaces the DarkNet53 network in the YOLOv3 algorithm with the CSDenseNet network, adds the SPPNet, PANet and channel attention mechanism CBAM structures in the multiscale detection module, and replaces the ordinary 3 × 3 convolution with 3 × 3 depth separable convolution. the YOLOv3 network structure and the S-MGDM network structure proposed in this study are shown in Fig. 10a,b. MGDM network structure is shown in Fig. 10a,b. In the S-MGDM network, after the input image is feature extracted by the CSDenseNet network, three scale feature maps with 256 resolutions of 52 × 52 pixels, 512 resolutions of 26 × 26 pixels and 1024 resolutions of 13 × 13 pixels are output; after the three feature maps obtained in the backbone feature extraction network, CBAM is added respectively, and the added The 13 × 13 × 1024 feature maps of CBAM have connected to SPPNet afterward, and finally these three feature maps are passed into PANet for enhanced feature extraction, and finally three feature maps feat1, feat2, and feat3 are obtained to output the prediction results.

The detection of the YOLO series consists of two phases, training and testing. In the training phase, the S-MGDM network model takes the information between the prediction frame and the true marker frame to create a loss function and is trained to minimise the loss functions. In the testing phase, the model predicts each input image of the test set, and the output, includes both target class and confidential information to complete the task of detecting the Shine-Muscat Grape target.

### Experimental platform and model parameter settings

The computer configuration used in this experiment is Intel(R) Core (TM) i5-7300HQ CPU @2.50 GHz, NVIDIA GeForce GTX1050, 8G running memory. The operating system used is windows 10, programming language Python 3.7, and deep learning framework Tensorflow 2.2, NVIDIA 472.12 driver, CUDA and CUDNN versions 10.1 and 7.6.5 respectively.

In the model training phase, nine anchor frames were reselected using the k-means clustering algorithm based on this dataset, with anchor frame sizes of 17 × 51, 32 × 71, 39 × 103, 53 × 143, 68 × 77, 91 × 111, 116 × 212, 133 × 134 and 243 × 331, and the model training parameters were consistent with yolov3, using the momentum The model is trained using the stochastic gradient (SGD + Momentum) optimizer with a momentum size of 0.9 and a decay coefficient of 0.0005 and a Batch Size of 4. The first two Resblock structures in the network model are modified according to the yolov3 pre-training weights for weight initialization, and 500 epochs of training iterations are trained using migration learning. The first 50 epochs were first frozen for fine-tuning the network, and their learning rate was set to 0.001; then the remaining 450 epochs were unfrozen for training, and their learning rate was set to 0.0001, and the learning rate was updated using cosine annealing^36^ decay, with the number of target categories being 1 and the labels being clusters, using Mosaic data augmentation, batch normalization, all the standard stuff, and multiscale training, the size of the input image is selected randomly from the multiscale set [320, 352,…,608] for multiscale training. In the test phase, the resolution size of the input image is 416 × 416 and the threshold of IoU is set to 0.5.

### Model evaluation indicators

The evaluation metrics used in this experiment are the F1 score^37^, which is commonly used in target detection, the Average Precision (AP), the number of floating-point operations (FLOPs)^38^, the speed metric FPS for evaluating target detection, and the network structure size as the evaluation metrics of the model. Among them, the F1 score, also known as the balanced F score, has a value size related to the accuracy P (Precision) and the recall R (Recall), which are considered in combination and are a kind of weighted average of the model accuracy and recall. The formulae for calculating the accuracy P, recall R, and F1 score are shown below.

4 $$ P = \frac{TP}{{TP + FP}} $$

5 $$ R = \frac{TP}{{TP + FN}} $$

6 $$ F1 = 2 \times \frac{P \times R}{{P + R}} $$

where TP (True Positive) indicates the number of target frames for which the model predicts a positive sample and is positive; FP (False Positive) indicates the number of target frames for which the model predicts a positive sample and is negative, and FN (False Negative) indicates the number of target frames for which the model predicts a negative sample and is positive.

The results of AP calculation are related to the accuracy P and recall R. The P–R curve can be drawn based on the multiple P and R values obtained when the threshold is equal to a certain value, and then the area contained under the P-R curve, i.e., the AP value, can be calculated by the formula. The calculation formula is as follows.

7 $$ AP = \sum\limits_{i}^{n} {\left( {R_{i} - R_{i - 1} } \right)} \max \left[ {P_{i} ,P_{i + 1} } \right] $$

where $n$ denotes the number of recalls; $R_{i}$ denotes the $i$-th ($i \in [1,n]$) recall.

### Involving plant research

Study on plant complies with relevant institutional, national, and international guidelines and legislation.

## Results and analysis

### Analysis of ablation experimental results of S-MGDM model

Ablation experiments^39^ refer to a series of experiments to verify the effectiveness of each improvement strategy on the target detection model. To verify the improvement of the effectiveness of each improvement method used in this model, this study tested 240 images from the test set incrementally in terms of the backbone feature extraction network, the multiscale detection module, and the loss function, respectively, and the results are shown in Table 3. Model A represents the original YOLOv3 detection algorithm; model B represents the addition of CSPNet structure as the backbone feature extraction structure of the network on top of the original YOLOv3; model C represents the fusion of DenseNet as the backbone network on top of model B; model D represents the addition of SPPNet structure to the output part of the backbone network on top of model C. Model E indicates that based on model D, the attention mechanism CBAM is added before the feature map is passed into the enhanced feature extraction network; model F indicates that based on model E, the PANet structure is added to the multiscale detection module and the ordinary convolution is replaced by the deep separable convolution; model G indicates that based on model F, the loss function in the original YOLOv3 is replaced by the CIOU loss function. According to Table 3, the floating point FLOPs of the S-MGDM network model proposed in this study are the smallest among all compared models, which is $3.216 \times 10^{10}$; the network structure is also only 62.2 MB, and the F1 score is the same as model F (0.91), while the average accuracy reaches the highest 96.73%; each structure added to the network model has a significant effect on improving the accuracy of identifying grapes, and the experimental The results show that the network model proposed in this study better balances the average detection accuracy, F1 score, floating point operations FLOPs, network structure size, and detection speed.

**Table 3:** Results of ablation experiments for each model.

Models	Model abbreviation	FPS	F1 score	AP/%	FLOPs/× 10^10^	MB
YOLOv3 DarkNet53	A	10.46	0.88	92.86	6.529	235
YOLOv3 DarkNet53 + CSPNet	B	15.78	0.90	93.52	5.960	213
B + DenseNet	C	18.76	0.91	93.03	4.846	208
C + SPPNet	D	18.21	0.89	94.26	4.943	210
D + CBAM	E	18.06	0.90	94.53	5.006	211
E + PANet	F	26.95	0.91	95.72	3.224	62.2
F + CIOU_Loss_	G	26.95	0.91	96.73	3.216	62.2

**Figure 11:**
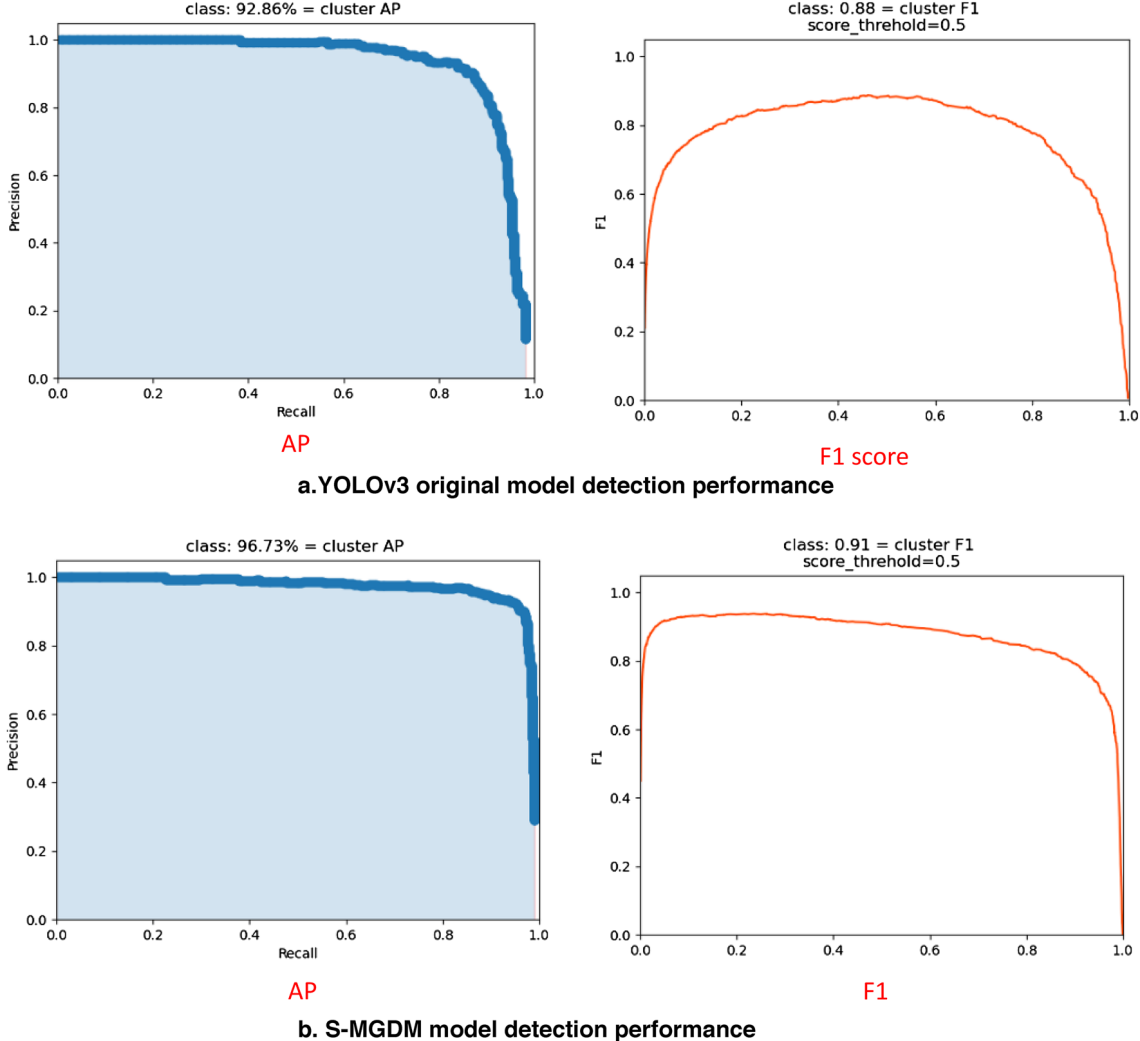
Performance visualization results of the original YOLOv3 model and the improved S-MGDM model.

**Figure 12:**
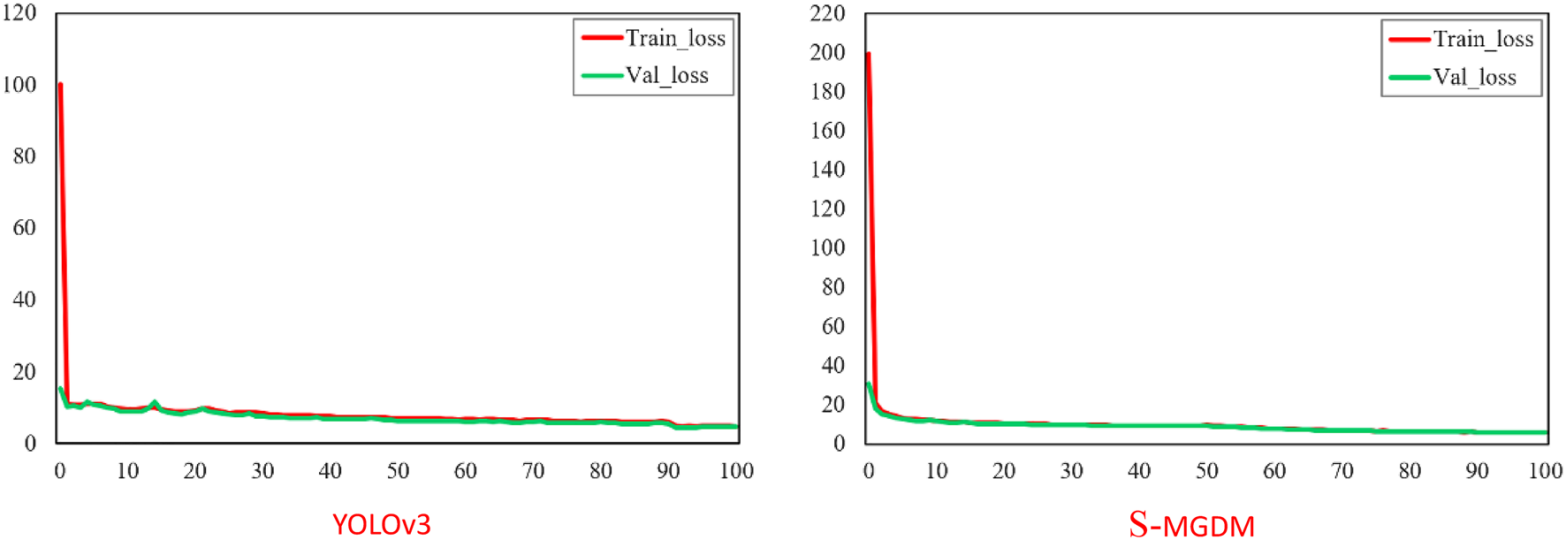
Loss curves of the original YOLOv3 model and the improved S-MGDM model.

Figure 11 shows the visualization results of the detection performance of the YOLOv3 model and the S-MGDM model proposed in this study, including the AP value and F1 score graphs. From Fig. 11, it can be found that the AP value and F1 score of the original YOLOv3 are 92.86% and 0.88, respectively, and the AP value and F1 score of the improved S-MGDM model are 96.73% and 0.91, respectively. They improved by 3.87 percentage points and 0.03, respectively, and the model performance was improved with the reduced amount of model parameters. The training loss curves of YOLOv3 and S-MGDM are shown in Fig. 12, and the loss values are recorded every 5 epochs for a total of 100 times. From Fig. 12, we can see that the training and validation losses of the improved S-MGDM model are lower than those of the YOLOv3 model, and the loss curves are smoother, indicating that the model has stronger feature extraction ability and better robustness, and the model training performance is better than that of the original model.

### Analysis of test results of different models

**Figure 13:**
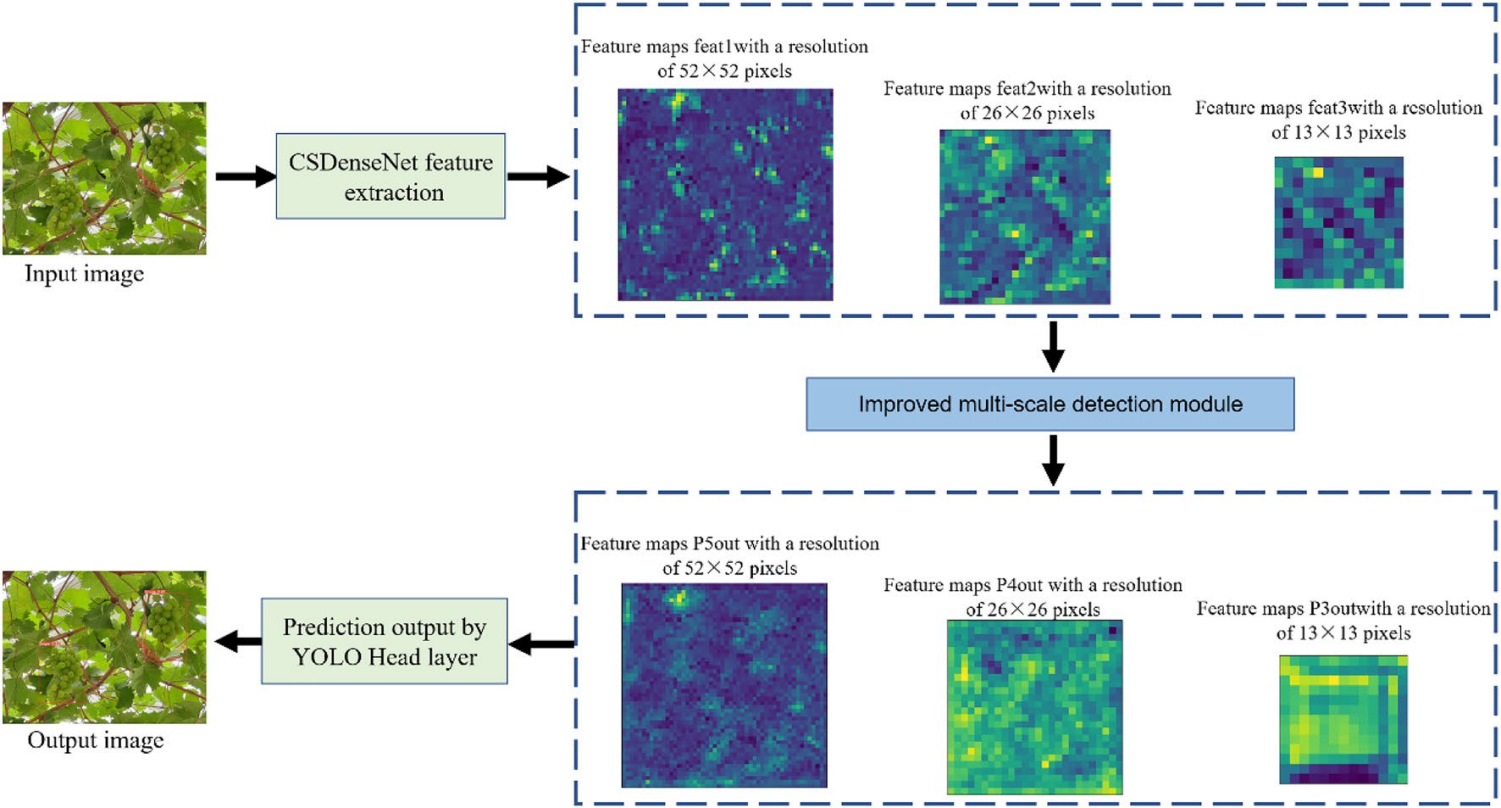
Flow chart for visualisation of the Shine-Muscat Grape image detection process under the S-MGDM model.

**Figure 14:**
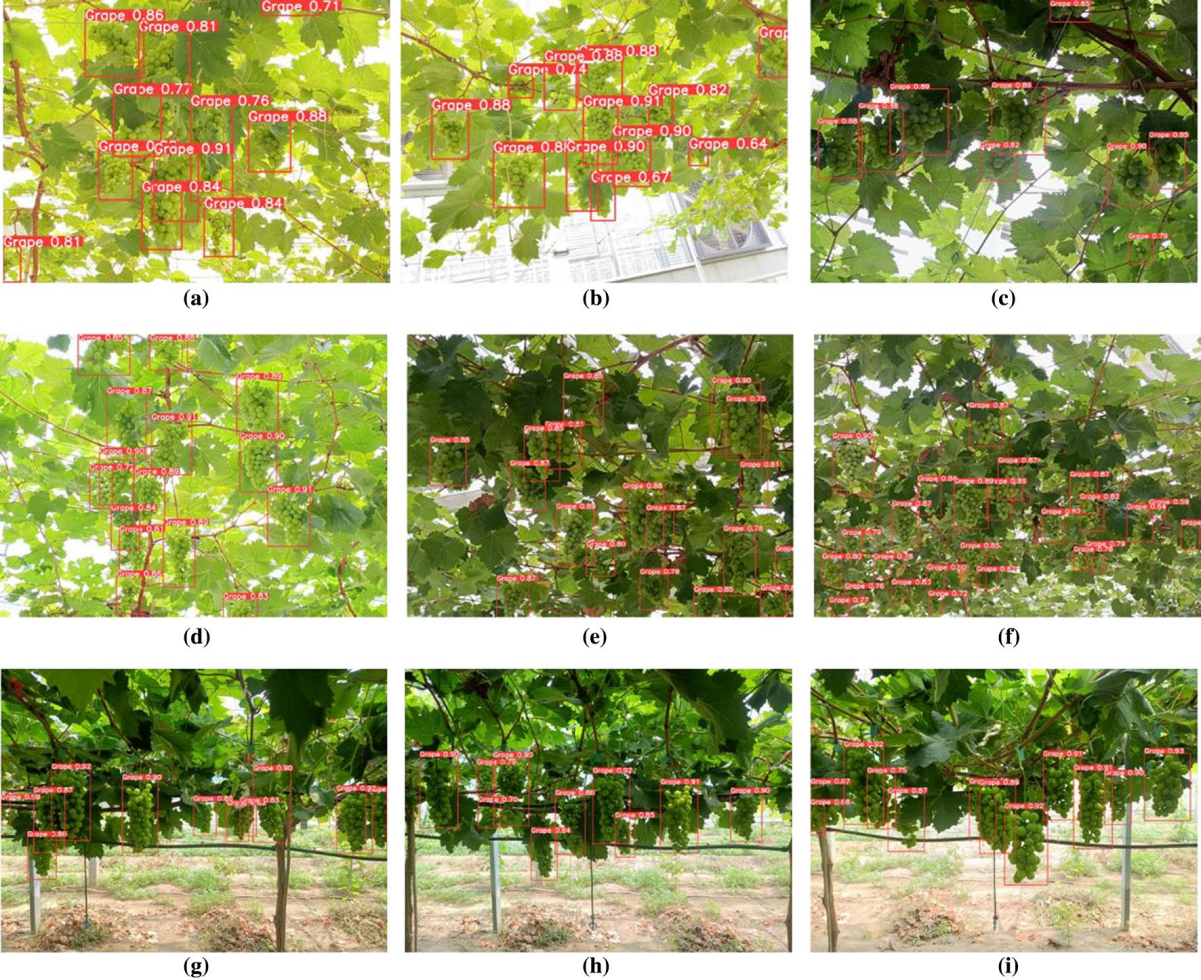
Partial detection results of the S-MGDM network model.

In the experimental stage, the improved S-MGDM network model proposed in this study was used to test using pictures from the Sunshine Rose grape test set, in which the visualization process for detecting Sunshine Rose is shown in Fig. 13, and some of the detection results under different environmental conditions are shown in Fig. 14. As can be seen from Fig. 13, the S-MGDM network performs initial feature extraction on the input sun rose grape image after the backbone feature extraction part, and it can be seen from the figure that the output three feature maps of different sizes contain only local feature information; then when these three feature maps are further feature extracted by the improved multi-scale detection module, the network will obtain global feature information (including the grape location information) at this time, and finally the prediction results containing target location, confidence and category are obtained after the YOLO Head layer. It is demonstrated that the S-MGDM network model proposed in this study can detect the Shine-Muscat Grape target accurately and quickly.

In order to verify the validity and feasibility of the proposed S-MGDM network model, the mainstream detection network model with the same evaluation index was selected for experiments based on a large number of references to the detection grape literature and under consistent experimental conditions. Five different models such as SSD^40^, YOLOv3, YOLOv4, and YOLOX-m^41^ were used to train and test the dataset, and the experimental results of their five detection models and the S-MGDM network model proposed in this study are shown in Table 4. As can be seen from Table 4, the S-MGDM model has the highest average detection accuracy of 96.73%, and the F1 score is second only to YOLOX-m with 91%, indicating that this model has a better balance of accuracy and recall; the YOLOX-m model has the highest recall and F1 score of 96.10% and 92%, respectively; the SSD model has the lowest accuracy of 82.14%, and the average detection accuracy is 87.78%, which is only higher than YOLOv4-tiny; YOLOv3 and YOLOv4 have higher detection accuracy with average detection accuracy of 92.86% and 94.51%, but their network structures are too large, 235 MB and 244 MB, respectively; YOLOv4-tiny model has the smallest network structure with only 22.4 MB, but its average detection accuracy is the worst, only 82.73%. Collectively, compared with several other models, the S-MGDM network model proposed in this study has a greater advantage in the performance of various evaluation indexes and can achieve the need for accurate and rapid detection of Shine-Muscat grapes in orchards.

**Table 4:** Comparison of evaluation indicators for different detection models.

Models	P/%	R/%	F1/%	AP/%	FLOPs/× 10^10^	MB	FPS
SSD^40^	82.14	82.44	82	87.78	6.283	90.7	19.73
YOLOv3^23^	91.22	85.66	88	92.86	6.529	235	20.46
YOLOv4^24^	92.07	88.30	90	94.51	5.960	244	19.69
YOLOv4-tiny^24^	83.51	77.00	80	82.73	0.679	22.4	78.07
YOLOX-m^41^	88.04	96.10	92	93.20	7.337	97.3	23.20
S-MGDM	95.66	85.96	91	96.73	3.216	62.2	26.95

The detection results of SSD, YOLOv3, YOLOv4, YOLOv4-tiny, YOLOX-m and S-MGDM network models experimentally compared in this study for the image with the file name test1.jpg (image 1) and the image with the file name test2.jpg (image 2) are shown in Table 5. As can be seen from Table 5, among the detection results of these two images, the YOLOv4-tiny model can correctly detect the least number of grapes, which is 18 bunches; the YOLOv4 detection model correctly detects more bunches, misses 2 bunches, and has better detection results, which is 22 bunches; although the S-MGDM detection model proposed in this study misses one bunch of grapes, it correctly detects the most number of grapes, which is as high as 23 bunches, and there is no false detection phenomenon in this detection model, which indicates that this model has better results in detecting grapes.

**Table 5:** Comparison of detection results for different detection models.

Models	Grape detection results of image 1			Grape detection results of image 2			Grape detection results of image 1 and image 2		
	Number of correct detection	Number of incorrect detection	Number of missing detection	Number of correct detection	Number of incorrect detection	Number of missing detection	Number of correct detection	Number of incorrect detection	Number of missing detection
SSD^40^	10	0	2	9	1	2	19	1	4
YOLOv3^23^	11	1	0	10	1	1	21	2	1
YOLOv4^24^	12	0	0	10	0	2	22	0	2
YOLOv4-tiny^24^	9	1	2	9	2	1	18	2	4
YOLOX-m^41^	11	0	1	10	2	0	21	2	1
S-MGDM	12	0	0	11	0	1	23	0	1

## Conclusion and future direction

1. We propose an S-MGDM network model (Shine-Muscat Grape Detection Model, S-MGDM) based on improved YOLOv3 for accurate and fast detection of Shine-Muscat Grapes. By using DarkNet53 to fuse CSPNet and DenseNet on the input images for initial extraction of richer underlying information of grapes. Add the attention mechanism CBAM in the multi-scale detection module to realize the network adaptive attention, which makes the neural network pay more attention to the channel and spatial information of grape pictures; then fuse the SPPNet structure and PANet structure, replace some of the convolution with depth separable convolution to realize the process of repeatedly extracting grape features to reduce the computation of network parameters and reduce the size of the network structure; finally The localization loss function is changed to CIOU loss function, which integrates the distance between the target and the bounding box, overlap rate and other factors to make the network prediction of grape target box regression become more stable and improve the fruit recognition accuracy.
2. Our proposed S-MGDM network model obtained 96.73% average accuracy on the Sunshine Rose grape test set with an F1 score of 91%, a target detection speed FPS of 26.95 frames per second, FLOPs of $3.216 \times {10}^{{{10}}}$, a network structure size of 62.2 MB, a network structure smaller than SSD, YOLOv3, YOLOv4 and YOLOX-m, and an F1 score and the average accuracy is higher than YOLOv4-tiny. comparing the results, our proposed S-MGDM network model structure indicates the best comprehensive detection performance.
3. Through the above experiments, it is shown that the S-MGDM network model proposed in this study has an excellent detection effect on ripe Shine-Muscat grapes, and can better identify and detect grape from branches and leaves with a similar color to the fruit, and the model is general and portable, and can also be used for other kinds of grape detection. There are some working ways to further improve our method as well, such as using image processing methods to remove distracting factors such as branches and leaves before inputting into the network, which in turn can improve the detection effect of the model.
4. In this paper, the accurate identification of Shine-Muscat grapes was achieved. Future work will transplant the model to the detection of other kinds of grapes and advance the deployment of the model in a picking robot, which will acquire the 3D coordinates of the grapes through the ROS system combined with binocular vision cameras and then control the robot arm to pick the grapes, aiming to create a grape picking robot.

## Data Availability

The data supporting this study’s findings are available from the corresponding author upon reasonable request.
